# The clinical significance of hyperthermic intraperitoneal chemotherapy combined with PD-1 inhibitor and systemic chemotherapy for advanced gastric cancer patients with peritoneal metastasis: a single-center retrospective study

**DOI:** 10.3389/fonc.2025.1728724

**Published:** 2026-01-12

**Authors:** Meijin Ren, Jiaqi Xie, Jingxin Liu, Yibing Wang, Ziyue Xiang, Shanping Li, Xiaoyan Yang, Naiqing Ding, Yang Yang

**Affiliations:** 1 Department of Oncology, Nanjing Drum Tower Hospital Clinical College of Nanjing University of Chinese Medicine, Nanjing, China; 2 Nanjing Drum Tower Hospital, Affiliated Hospital of Medical School, Nanjing University, Nanjing, China

**Keywords:** gastric cancer (GC), hyperthermic intraperitoneal chemotherapy (HIPEC), immune checkpoint inhibitors (ICI), peritoneal metastasis (PM), systemic chemotherapy

## Abstract

**Background:**

Peritoneal metastasis (PM) in gastric cancer (GC) correlates with a poor prognosis. PD-1 inhibitor has significantly transformed the treatment landscape for GC, but data on the combination of HIPEC with PD-1 inhibitor and systemic chemotherapy are limited. This study evaluated the efficacy and safety of this triplet therapy as first-line treatment for GC with PM.

**Methods:**

This was a retrospective, single-center study that included 34 advanced GC patients with PM. All patients received HIPEC combined with PD-1 inhibitor and systemic chemotherapy as first-line treatment (Nov 2021-Jun 2024). Primary endpoints were progression-free survival (PFS) and the duration of ascites control. Secondary endpoints were overall survival (OS) and adverse events (AEs).

**Results:**

Median PFS was 7.8 months (range: 0.8-20.8), median OS was 14.3 months (range: 1.7-23.9). Patients diagnosed with moderate amount of ascites had significantly poorer PFS than with none or small amount of ascites (HR = 2.804, *p* = 0.0197). Univariable Cox regression analysis showed that age ≤ 60 years was associated with ascites progression (HR = 4.266, *p* = 0.02) and death (HR = 2.732, *p* = 0.04). Additionally, univariable Cox regression analysis showed that age ≤ 60 years (HR = 5.762, *p* = 0.001) and moderate amount of ascites (HR = 2.923, *p* = 0.027) were potential risk factors for disease progression. The most common adverse events were anemia, hypokalemia, thrombocytopenia and leukopenia. All adverse events were manageable.

**Conclusion:**

HIPEC combined with PD-1 inhibitor and systemic chemotherapy demonstrated encouraging survival and ascites control with an acceptable safety profile in GC patients with PM.

## 1 Introduction

GC is one of the most prevalent malignant tumors worldwide, with its incidence and mortality rates ranking fifth on a global scale. PM is the most frequent distant site of metastasis in advanced GC and is one of the primary factors contributing to poor prognosis. PD-1 inhibitors have revolutionized the treatment of GC in recent years, extending the overall survival time of patients with advanced GC to approximately 14 months (1). The combination of chemotherapy and immunotherapy has achieved remarkable success in advanced GC and has been established as a standard first-line treatment regimen (1). Nonetheless, its effectiveness is constrained in patients with PM. This limitation can be attributed to several factors, including the ‘peritoneal plasma barrier’ that impedes drug penetration, an immunosuppressive microenvironment, and the persistence of free cancer cells within the peritoneal cavity (2, 3).

Based on the results of the DRAGON-01 trial (4), the Chinese Society of Clinical Oncology (CSCO) Guidelines for Gastric Cancer (5) have added “systemic chemotherapy plus intraperitoneal chemotherapy” as a Grade I recommendation for the treatment of peritoneal metastasis. This inclusion provides a new standard treatment option for GC patients with PM. HIPEC represents a localized treatment modality that entails the infusion of chemotherapeutic agents into the peritoneal cavity at a controlled temperature and circulation duration (6). This approach capitalizes on the synergistic interactions of chemotherapy, hyperthermia, and mechanical lavage to target and eradicate free-floating cancer cells, subclinical lesions, and micro-metastases measuring less than 3 mm, thereby addressing and preventing PM associated with malignant tumors. Hyperthermia is known to alter tumor physiology and improve chemotherapy efficacy (7). Furthermore, evidence indicates that hyperthermia can trigger immunogenic cell death (ICD) in tumor cells, leading to the release of damage-associated molecular patterns (DAMPs), such as heat shock proteins 70/90 (HSP70/90), high mobility group box 1 (HMGB1), and adenosine triphosphate (ATP). These released DAMPs can enhance the antitumor efficacy of PD-1 inhibitors by stimulating dendritic cell antigen presentation and promoting CD8+ T cell infiltration (8). However, there are few studies on the combination of HIPEC with PD-1 inhibitor and systemic chemotherapy for the treatment of GC with PM. The main purpose of this study was to assess the prognosis and safety of HIPEC combined with PD-1 inhibitor and systemic chemotherapy in the first-line treatment of advanced GC patients with PM and to explore the factors affecting patients’ prognosis.

## 2 Patients and methods

### 2.1 Patients

This study was approved by the Institutional Review Board of Nanjing Drum Tower Hospital of Nanjing University Medical School (IRB number: 2025-0160-02, date of approval: April 27, 2025). Informed consent was obtained from all participants, which included consent for the publication of images and clinical details. This was a retrospective, single-center study. The inclusion criteria included: patients diagnosed with advanced GC with PM by surgical exploration, imaging, and histology; patients who received HIPEC combined with PD-1 inhibitor and systemic chemotherapy as the first-line treatment; patients with complete medical history and laboratory parameters. The exclusion criteria were as follows: received other anti-tumor treatments within 6 months before inclusion in the study, severe infection, vital organ insufficiency, coagulation dysfunction, and patients with unstable vital signs. 34 patients were finally enrolled in this study.

### 2.2 Procedures

All patients received HIPEC treatment. Two inflow and two outflow catheters were inserted into the abdominal cavity. The pre-heated 0.9% sodium chloride containing cisplatin was infused into the peritoneal cavity using a closed-circuit hyperthermic intraperitoneal perfusion system (BR-TRG-III type). The total dose of cisplatin per session was 40 mg/m² body surface area. Of this total, 20 mg was added to the perfusate for circulation. The perfusate was maintained at 41-43 °C and circulated with a flow rate of 400–500 ml/min for 60 minutes. The perfusate was drained from the peritoneal cavity after completion of the HIPEC procedure. the remainder of the cisplatin dose was instilled into the peritoneal cavity for retention.

Each treatment cycle comprised two HIPEC sessions delivered 24–48 hours apart. The exact dose for each session was determined beforehand based on the patient’s most recent clinical parameters, including age, body surface area, renal function, and performance status. All patients received a standardized renal protection protocol involving proactive hydration, electrolyte management, and close monitoring. No dose reductions between sessions/cycles were required due to renal toxicity. Systemic antitumor therapy commenced 24 hours after HIPEC completion.

### 2.3 Follow-up

Survival data were collected via structured telephone interviews, capturing the interval from initial HIPEC administration to endpoint events (disease progression or death). The final follow-up cutoff date was September 25, 2024.

### 2.4 Response evaluation

The study’s primary endpoints were PFS and ascites control duration, and the secondary endpoints were OS and AEs. OS was defined as the interval from the first HIPEC to all-cause mortality or last follow-up. PFS was defined as the interval from the first HIPEC to radiologic/clinical progression or last follow-up. Time to ascites control was defined as the interval from the first HIPEC to ascites progression or last follow-up.

Objective efficacy was evaluated according to RECIST 1.1, including complete response (CR), partial response (PR), stable disease (SD), and progressive disease (PD). The objective response rate (ORR) was defined as the sum of the proportions of complete and partial response. The amount of ascites was evaluated by computed tomography or abdominal ultrasound and categorized as none, small (within the pelvic cavity), or moderate (beyond the pelvic cavity) at enrollment. The efficacy of ascites control was evaluated by computed tomography or abdominal ultrasound as follows: CR: complete disappearance of ascites for more than 4 weeks; PR: more than 50% reduction in ascites; SD: less than 50% reduction or no more than 25% increase in ascites; PD: more than 25% increase in ascites compared with pretreatment.

### 2.5 Statistical analysis

Analyses were performed using SPSS version 25.0 (IBM Corp., Armonk, NY, USA). Continuous variables are presented as median values (range), and categorical variables are represented as frequencies (%). Survival distributions were estimated using the Kaplan-Meier method, and comparisons between groups were carried out employing log-rank tests (significance threshold: *P* ≤ 0.05). Univariable Cox proportional hazards models were used to assess prognostic factors for PFS, OS, and the duration of ascites control. Results for all analyzed variables are reported as hazard ratios (HR) with corresponding 95% confidence intervals (9–11).

## 3 Results

### 3.1 Patient characteristics and treatments

A total of 34 patients were enrolled. Patient characteristics and treatments are presented in **Table 1**. The median age at the time of diagnosis was 59.0 years (range: 26–82 years). There were 16 (47.06%) women and 18 (52.94%) men. Histologically, 22 (64.71%) patients were diagnosed with tubular adenocarcinoma, while 12 (35.29%) patients exhibited either mucinous adenocarcinoma or signet-ring cell carcinoma. Notably, 31 (91.18%) patients presented with tumors classified as poorly differentiated. All patients enrolled were in the advanced stage of GC, including 21 (61.76%) with PM and 13 (38.24%) with metastasis to both the peritoneum and retroperitoneal lymph nodes. 16 (47.06%) patients presented with none or small amount of ascites, whereas 18 (52.94%) patients exhibited moderate amount of ascites at the time of enrollment. The majority of the enrolled patients exhibited low programmed death-ligand 1 (PD-L1) expression [combined positive score (CPS) <5], and negative human epidermal growth factor receptor 2 (HER2) status.

**Table 1:** Patient characteristics and treatments.

Variables	n (%)
Age, median (range), years	59 (26–82)
Gender	
Male	18 (52.94)
Female	16 (47.06)
Histology	
Tubular adenocarcinoma	22 (64.71)
Mucinous adenocarcinoma and signet-ring cell carcinoma	12 (35.29)
Differentiation degree	
Poorly differentiated	31 (91.18)
Moderately-poorly differentiated	3 (8.82)
Primary tumor location	
Body	21 (61.76)
Pylorus	8 (23.53)
Cardia	5 (14.71)
Metastatic organs	
Peritoneum	21 (61.76)
Peritoneum and Retroperitoneal lymph nodes	13 (38.24)
T stage	
cT3	13 (38.23)
cT4a	11 (32.35)
cT4b	7 (20.59)
cTx	3 (8.82)
N stage	
N2	6 (17.65)
N3	23 (67.65)
Nx	5 (14.71)
CPS score	
CPS < 5	21 (61.76)
CPS ≥ 5	5 (14.71)
Unknown	8 (23.53)
HER2 status	
Negative	29 (85.29)
Positive	3 (8.82)
Unknown	2 (5.88)
TMB	
TMB-L	12 (35.29)
TMB-H	1 (2.94)
Unknown	21 (61.76)
MSI	
MSI-L	1 (2.94)
MSS	10 (29.41)
Unknown	23 (67.64)
Prior gastrectomy	
Distal	1 (2.94)
Total	2 (5.88)
Amount of ascites*	
None	10 (29.41)
Small	6 (17.65)
Moderate	18 (52.94)
Number of HIPEC cycles	
1	28 (82.35)
2	6 (17.65)
The first cycle of HIPEC medication	
Cisplatin	34 (100.00)
The second cycle of HIPEC medication	
Cisplatin	6 (100.00)
Systemic treatment	
PD-1 inhibitors +Oxaliplatin +Fluorouracil	31 (91.18)
PD-1 inhibitors +Trastuzumab +Oxaliplatin +Fluorouracil	3 (8.82)

CPS, Combined Positive Score; TMB, Tumor Mutational Burden; TMB-H, TMB-High; TMB-L, TMB-Low; MSI, Microsatellite Instability; MSI-L, MSI-Low; MSS, Microsatellite Stable; HIPEC, Hyperthermic Intraperitoneal Chemotherapy; PD-1, Programmed cell death protein 1

*Evaluated by computed tomography: small, within the pelvic cavity; moderate, beyond the pelvic cavity

3 of the 34 patients had previously undergone radical surgery for GC and subsequently developed PM two years later. The remaining 31 patients were inoperable due to extensive PM at the time of initial diagnosis. In terms of medication, all patients received HIPEC in conjunction with PD-1 inhibitor (including sintilimab, nivolumab, and tislelizumab) and systemic chemotherapy as first-line treatment. Following six cycles of systemic chemotherapy, eight patients received maintenance immunotherapy combined with oral chemotherapy until disease progression or study completion. Cisplatin was selected as the HIPEC agent based on its favorable intraperitoneal pharmacokinetics, proven thermosynergy, and extensive clinical experience in the management of peritoneal metastases from gastrointestinal cancers (9–11). Additionally, three patients who were HER2-positive received supplementary anti-HER2 therapy.

**Figure 1:**
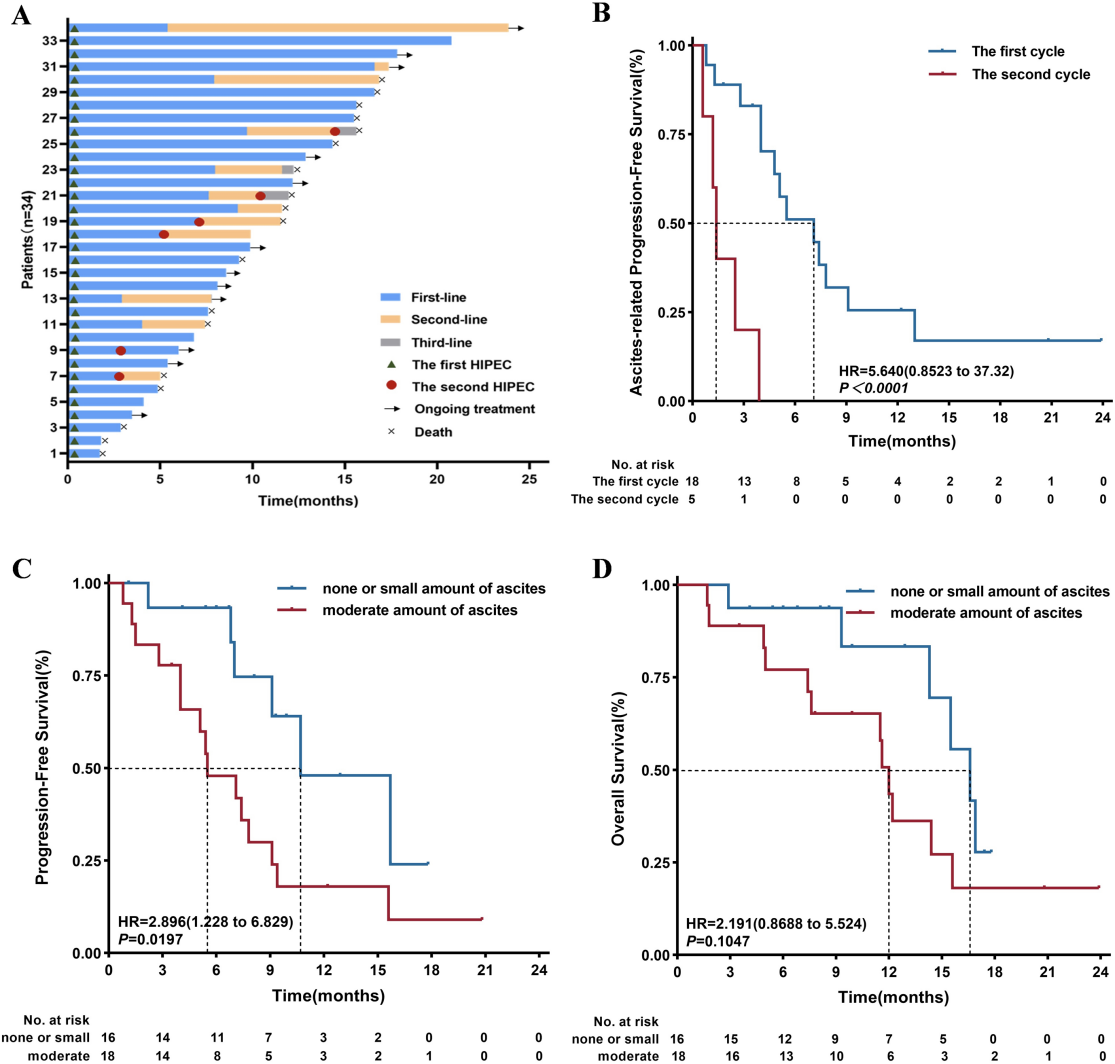
**(A)** The Swimmer plot of treatment duration (n=34) demonstrated treatment lines, HIPEC cycles, ongoing treatment, and mortality. **(B)** The second cycle of HIPEC demonstrated significantly inferior ascites control efficacy compared to the first cycle (HR = 54.70, 95% CI: 7.417-403.4, *P* < 0.0001). **(C)** Patients diagnosed with moderate amount of ascites had significantly poorer PFS than those with none or small amount of ascites (HR = 2.896, 95% CI: 1.228-6.829; *P* = 0.0197). **(D)** Patients diagnosed with moderate amount of ascites had significantly poorer OS than those with none or small amount of ascites, though the difference was not statistically significant (HR = 2.191, 95% CI: 0.869-5.524; *P* = 0.1047). *Colored solid bars: treatment duration and number of treatment lines of patients; Triangle: the first HIPEC; Circle: the second HIPEC; Arrow: ongoing treatment; Cross: death*.

After the first round of treatment, three patients underwent gastrectomy after MDT discussion. A total of six patients (17.6%) received a second round of HIPEC combined with PD-1 inhibitor and systemic chemotherapy. Of these, five patients (14.7%) underwent retreatment due to the development of new ascites or disease progression. The remaining patient received a second HIPEC cycle because laparoscopic exploration did not meet the criteria for surgical intervention, despite imaging showing disease remission (**Figure 1A**).

### 3.2 Clinical outcomes

The median follow-up duration was 17.4 months. At the end of the study, 9 patients (26.5%) remained progression-free, 12 patients (35.3%) were alive, which included 3 (8.82%) and 5 (14.7%) from the subgroup with moderate amount of ascites at enrollment, respectively (**Figure 1A**). Survival outcomes were evaluated using Kaplan-Meier curves.

In a cohort of patients who presented with none or small amount of ascites at baseline (n=16), none developed new ascites during a median follow-up period of 8.0 months (range: 1.1-17.8 months). Among the 18 patients with moderate amount of ascites, sustained ascites control was achieved in 5 patients (27.8%) at the final follow-up, with a median follow-up period of 12.2 months (range: 1.8-23.9 months). The median duration of ascites control after the first and second cycles of HIPEC was 7.5 months (range: 0.8-23.9 months) and 1.4 months (range: 0.6-3.9 months), respectively (**Figure 1B**). At the first imaging follow-up, efficacy analysis of ascites control indicated a complete response rate of 50.0% (9/18), partial response of 33.3% (6/18), stable disease of 16.7% (3/18), and an overall response rate of 83.3%.

Systemic tumor control and survival outcomes were further evaluated in the entire cohort (n = 34). The median PFS and median OS were 7.8 months (range: 0.8-20.8 months) and 14.3 months (range: 1.7-23.9 months). Notably, patients who presented with none or small amount of ascites exhibited significantly prolonged median PFS compared to those with moderate amount of ascites (10.7 vs. 5.5 months, *p* < 0.05; **Figure 1C**). Although not statistically significant, a numerical trend toward improved OS was observed in the none or small amount ascites subgroup (16.6 vs. 12.0 months; *p* = 0.10; **Figure 1D**).

### 3.3 Prognostic factors

To investigate prognostic factors associated with treatment outcomes in advanced GC patients with PM from HIPEC combined with PD-1 inhibitor and systemic chemotherapy, baseline clinical characteristics and laboratory parameters were analyzed using univariable Cox regression. Cut-off values for neutrophil-to-lymphocyte ratio (NLR), platelet-to- lymphocyte ratio (PLR), C-reactive protein (CRP), and systemic immune-inflammation index (SII) were determined based on median values.

**Figure 2:**
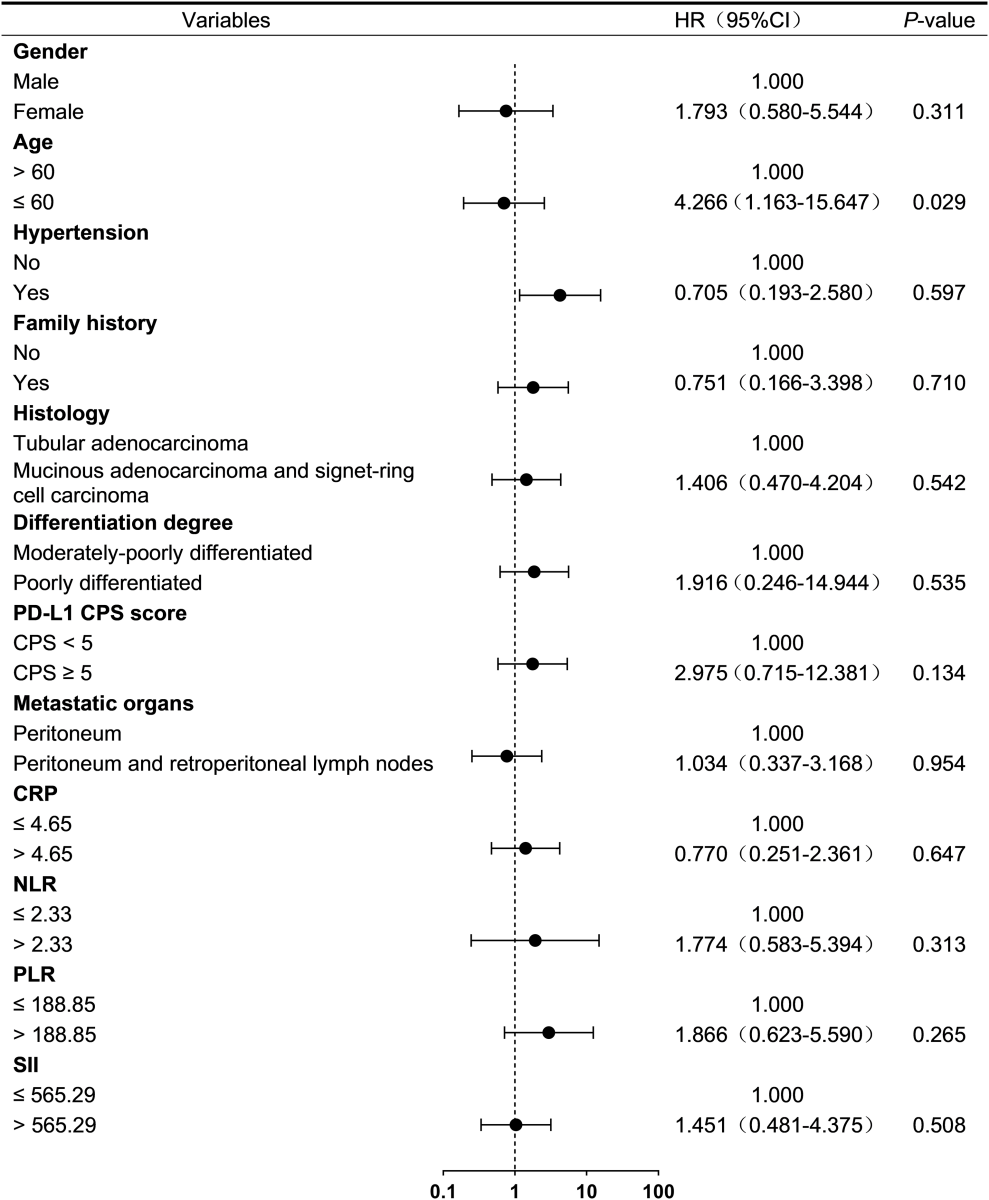
Univariate analysis of ascites-related progression-free survival. CPS, combined positive score; PD-1, programmed cell death protein 1; HER2, human epidermal growth factor receptor 2; CRP, C-reactive protein; NLR, neutrophil-to-lymphocyte ratio; PLR, platelet-to-lymphocyte ratio; SII, systemic immune-inflammation index.

**Figure 3:**
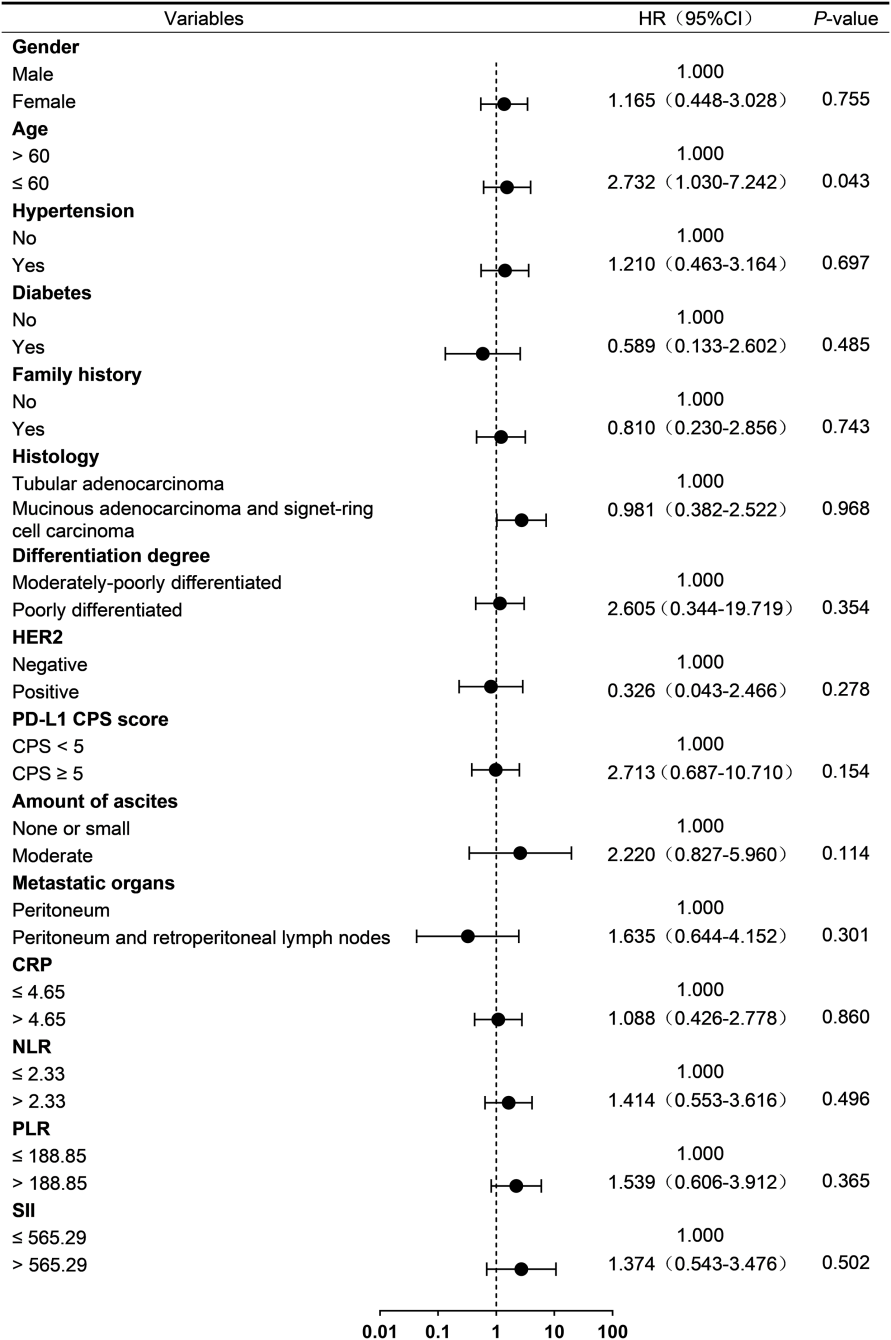
Univariate analysis of OS. CPS, combined positive score; PD-1, programmed cell death protein 1; HER2, human epidermal growth factor receptor 2; CRP, C-reactive protein; NLR, neutrophil-to-lymphocyte ratio; PLR, platelet-to-lymphocyte ratio; SII, systemic immune-inflammation index.

**Figure 4:**
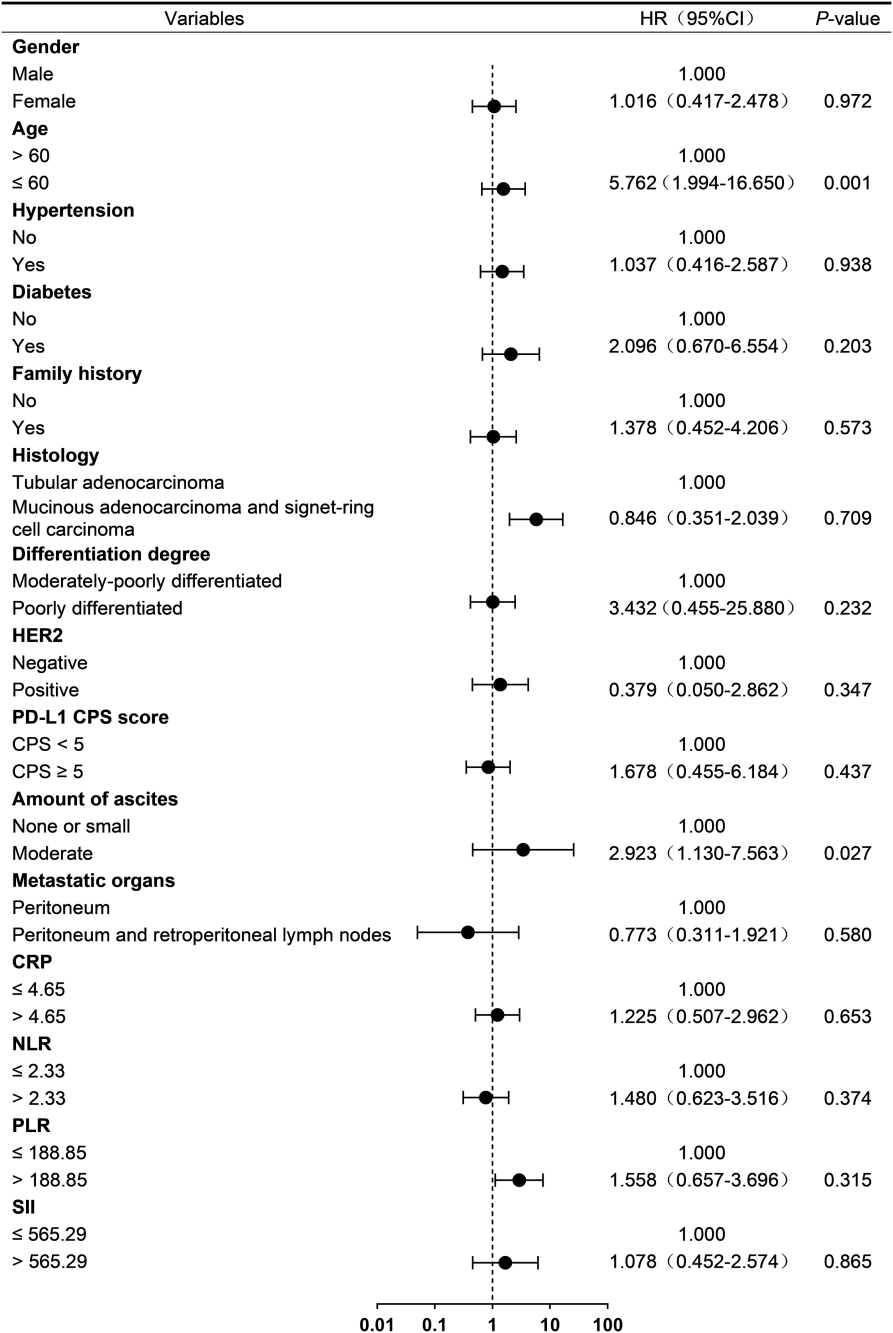
Univariate analysis of PFS. CPS, combined positive score; PD-1, programmed cell death protein 1; HER2, human epidermal growth factor receptor 2; CRP, C-reactive protein; NLR, neutrophil-to-lymphocyte ratio; PLR, platelet-to-lymphocyte ratio; SII, systemic immune-inflammation index.

In exploratory univariable analysis, age ≤ 60 years was associated with ascites progression (HR = 4.266, 95% CI: 1.163-15.647; *p* = 0.029; **Figure 2**) and death (HR = 2.732, 95% CI: 1.030-7.242; *p* = 0.043; **Figure 3**). Furthermore, age ≤ 60 years (HR = 5.762, 95% CI: 1.994-16.650; *p* = 0.00) and the presence of a moderate amount of ascites (HR = 2.923, 95% CI: 1.130-7.563; *p* = 0.02) were identified as potential risk factors for disease progression in this cohort. (**Figure 4**).

### 3.4 Safety analysis

Adverse events were graded according to the National Cancer Institute Common Terminology Criteria for Adverse Events (version 5.0), with the highest grade per event type recorded for each patient. Safety profiles are presented by treatment phase: during systemic therapy (**Table 2**), within 30 days of HIPEC (**Table 3**), and post-treatment (**Table 4**). Systemic therapy was typically initiated within 24 hours after HIPEC, resulting in an overlap between the “within 30 days of HIPEC” phase and the “systemic therapy” phase.

**Table 2:** Treatment-related adverse events occurring during systemic therapy.

Adverse event, n (%)	Total	Chemotherapy-related				Immunotherapy-related				HIPEC-related			
		G1	G2	G3	G4	G1	G2	G3	G4	G1	G2	G3	G4
Leukopenia	16 (47.1)	9 (26.5)	6 (17.7)	1 (2.9)	—	—	—	—	—	—	—	—	—
Neutropenia	13 (38.2)	6 (17.6)	6 (17.6)	1 (2.9)	—	—	—	—	—	—	—	—	—
Thrombocytopenia	20 (58.8)	10 (29.4)	6 (17.6)	4 (11.8)	—	—	—	—	—	—	—	—	—
Anemia	33 (97.1)	23 (67.6)	8 (23.5)	2 (5.9)	—	—	—	—	—	—	—	—	—
Nausea	10 (29.4)	8 (23.5)	2 (5.9)	—	—	—	—	—	—	—	—	—	—
Vomiting	8 (23.5)	6 (17.6)	2 (5.9)	—	—	—	—	—	—	—	—	—	—
Diarrhea	1 (2.9)	1 (2.9)	—	—	—	—	—	—	—	—	—	—	—
Hyponatremia	12 (35.3)	12 (35.3)	—	—	—	—	—	—	—	—	—	—	—
Hypokalemia	21 (61.8)	19 (55.9)	2 (5.9)	—	—	—	—	—	—	—	—	—	—
Creatinine increased	1 (2.9)	1 (2.9)	—	—	—	—	—	—	—	—	—	—	—
Peripheral sensory neuropathy	4 (11.8)	4 (11.8)	—	—	—	—	—	—	—	—	—	—	—
AST increased	11 (32.4)	9 (26.5)	2 (5.9)	—	—	—	—	—	—	—	—	—	—
ALT increased	11 (32.4)	11 (32.4)	—	—	—	—	—	—	—	—	—	—	—
Blood bilirubin increased	7 (20.6)	5 (14.7)	1 (2.9)	—	—	—	1 (2.9)	—	—	—	—	—	—
Skin rash	8 (23.5)	—	—	—	—	6 (17.6)	1 (2.9)	1 (2.9)	—	—	—	—	—
Hypothyroidism	4 (11.8)	—	—	—	—	3 (8.8)	1 (2.9)	—	—	—	—	—	—
Fever	8 (23.5)	—	—	—	—	1 (2.9)	—	—	—	7 (20.6)	—	—	—
Infection	3 (8.8)	—	—	—	—	—	—	—	—	2 (5.9)	1 (2.9)	—	—
Abdominal pain	12 (35.3)	—	—	—	—	—	—	—	—	12 (35.3)	—	—	—

HIPEC, Hyperthermic Intraperitoneal Chemotherapy; AST, aspartate aminotransferase; ALT, alanine aminotransferase

The severity grade for each AE type was recorded as the highest grade experienced by the patient during systemic therapy period.

Data are presented as the number of patients (%), based on the total cohort (n=34).

**Table 3:** Treatment-related adverse events occurring within 30 days of HIPEC.

Adverse event, n (%)	Total	Chemotherapy-related				Immunotherapy-related				HIPEC-related			
		G1	G2	G3	G4	G1	G2	G3	G4	G1	G2	G3	G4
Leukopenia	5 (14.7)	3 (8.8)	2 (5.9)	—	—	—	—	—	—	—	—	—	—
Neutropenia	2 (5.9)	2 (5.9)	0 (0.0)	—	—	—	—	—	—	—	—	—	—
Thrombocytopenia	9 (26.5)	7 (20.6)	1 (2.9)	1 (2.9)	—	—	—	—	—	—	—	—	—
Anemia	24 (70.6)	18 (52.9)	5 (14.7)	1 (2.9)	—	—	—	—	—	—	—	—	—
Nausea	7 (20.6)	6 (17.6)	1 (2.9)	—	—	—	—	—	—	—	—	—	—
Vomiting	3 (8.8)	3 (8.8)	—	—	—	—	—	—	—	—	—	—	—
Diarrhea	2 (5.9)	2 (5.9)	—	—	—	—	—	—	—	—	—	—	—
Hyponatremia	8 (23.5)	8 (23.5)	—	—	—	—	—	—	—	—	—	—	—
Hypokalemia	10 (29.4)	10 (29.4)	—	—	—	—	—	—	—	—	—	—	—
Creatinine increased	—	—	—	—	—	—	—	—	—	—	—	—	—
Peripheral sensory neuropathy	—	—	—	—	—	—	—	—	—	—	—	—	—
AST increased	3 (8.8)	3 (8.8)	—	—	—	—	—	—	—	—	—	—	—
ALT increased	4 (11.8)	4 (11.8)	—	—	—	—	—	—	—	—	—	—	—
Blood bilirubin increased	3 (8.8)	3 (8.8)	—	—	—	—	—	—	—	—	—	—	—
Skin rash	—	—	—	—	—	—	—	—	—	—	—	—	—
Hypothyroidism	—	—	—	—	—	—	—	—	—	—	—	—	—
Fever	7 (20.6)	—	—	—	—	—	—	—	—	7 (20.6)	—	—	—
Infection	3 (8.8)	—	—	—	—	—	—	—	—	2 (5.9)	1 (2.9)	—	—
Abdominal pain	12 (35.3)	—	—	—	—	—	—	—	—	12 (35.3)	—	—	—

HIPEC, Hyperthermic Intraperitoneal Chemotherapy; AST, aspartate aminotransferase; ALT, alanine aminotransferase

The severity grade for each AE type was recorded as the highest grade experienced by the patient during the 30-day observation period following HIPEC.

Data are presented as the number of patients (%), based on the total cohort (n=34).

**Table 4:** Patients with key adverse events by treatment phase.

Adverse event	Within 30 days of HIPEC, n (%)	During systemic therapy, n (%)	Post-treatment, n (%)
Any grade ≥a events	2 (5.9)	6 (17.6)	0 (0.0)
Any chemotherapy-related	28 (82.4)	33 (97.1)	0 (0.0)
Any immunotherapy-related	0 (0.0)	13 (38.2)	1 (2.9)
Any HIPEC-related	12 (35.3)	12 (35.3)	0 (0.0)

HIPEC, Hyperthermic Intraperitoneal Chemotherapy.

Data are presented as the number of patients (%), based on the total cohort (n=34).

“Any” refers to the proportion of patients who experienced at least one event within the specified category during the phase.

During systemic therapy, the most common all-grade adverse events were anemia (97.1%), hypokalemia (61.8%), thrombocytopenia (58.8%), and leukopenia (47.1%) (**Table 2**). Grade 3–4 adverse events (AEs) occurred in 6 patients (17.6%), primarily thrombocytopenia (11.8%) and anemia (5.9%) (**Tables 2**, **4**). Immune-related adverse events (irAEs) of any grade were observed, most commonly elevated skin rash (23.5%), and hypothyroidism (11.8%) (**Table 2**).

Within 30 days after HIPEC, the most common AEs were anemia (70.6%, 24/34), abdominal pain (35.3%, 12/34), hypokalemia (29.4%, 10/34), and thrombocytopenia (26.5%, 9/34) (**Table 3**). AEs specifically attributed to the HIPEC procedure occurred in 12 patients (35.3%), including abdominal pain (35.3%), fever (20.6%), and infection (8.8%) (**Tables 3**, **4**).

As summarized in **Table 4**, grade ≥3 AEs were concentrated during the systemic therapy phase (17.6%), Similarly, irAEs were reported exclusively during systemic therapy. Regarding management, 5 of the 6 patients with grade 3–4 AEs resumed their original systemic regimen within one week following supportive care. One patient discontinued immunotherapy due to a grade 3 rash and continued with chemotherapy alone. All AEs resolved with supportive interventions. No unexpected serious adverse events or treatment-related deaths were occurred.

## 4 Discussion

PM is a common manifestation of GC dissemination and significantly impacts the prognosis of patients with advanced-stage disease. The median survival of GC patients with PM has been reported to be 3–9 months, rarely exceeding 12 months even with standard systemic chemotherapy (12).

Conventional cytotoxic chemotherapy demonstrates limited efficacy in improving survival for GC with PM. HIPEC is recommended for GC patients with PM to reduce the tumor burden. Clinical studies have demonstrated that the combination of cytoreductive surgery (CRS) with HIPEC and systemic chemotherapy significantly prolongs OS compared to chemotherapy in advanced GC patients with PM (13). However, CRS is a high-risk surgical procedure, associated with high rates of complications and mortality, which significantly restricts its clinical application in GC with PM (14). A multicenter cohort study revealed that HIPEC combined with systemic chemotherapy provided statistically significant survival benefits compared with standard systemic chemotherapy for GC patients with PM, even if CRS is not performed (15). This finding suggests the potential clinical value of HIPEC-based regimens beyond the context of CRS. Further clinical investigations are warranted to optimize therapeutic protocols and validate the efficacy of HIPEC-based combinatorial regimens.

The advent of immunotherapy has expanded therapeutic horizons in oncology. The combination of chemotherapy and immunotherapy has significantly improved survival outcomes in patients with advanced GC (1). However, the survival benefits of this combined regimen in patients with PM remain controversial. Several phase III clinical trials have reported subgroup analyses of chemotherapy combined with PD-1 inhibitor as first-line therapy for GC patients with PM. A *post hoc* analysis of the RATIONALE-305 trial demonstrated that tislelizumab plus chemotherapy exhibited prolonged OS compared to chemotherapy alone in patients with PM [median OS: 12.3 months vs. 11.8 months; HR 0.78 (95% CI 0.64-0.96)] (16, 17). Contrasting these results, the ATTRACTION-4 trial reported inferior survival outcomes with nivolumab plus chemotherapy compared to chemotherapy alone (18). These findings suggest that the combination of PD-1 inhibitor and chemotherapy may confer potential clinical benefits for GC patients with PM. Furthermore, growing evidence highlights the synergistic potential of integrating locoregional intraperitoneal therapies with systemic immunotherapy (19, 20). The current PIANO trial (NCT03172416) indicates that the use of pressurized intraperitoneal aerosol chemotherapy with oxaliplatin (PIPAC-OX) in combination with nivolumab can boost immune activation and checkpoint inhibition. This approach presented a new framework for treating gastric peritoneal carcinomatosis.

The therapeutic efficacy of HIPEC combined with PD-1 inhibitor and systemic chemotherapy in our study for advanced GC with PM demonstrated superior survival outcomes compared to contemporary first-line therapy. The ATTRACTION-4 trial demonstrated that nivolumab combined with chemotherapy as first-line therapy for advanced or recurrent gastric/gastroesophageal junction (G/GEJ) cancer with PM achieved a median PFS of 7.46 months and a median OS of 13.67 months (18). A *post hoc* analysis of the RATIONALE-305 trial demonstrated a median OS of 12.3 months with tislelizumab plus chemotherapy as first-line therapy for G/GEJ cancer with PM (16, 17). Our cohort achieved a median OS of 14.3 months and median PFS of 7.8 months, these outcomes appear favorable compared to published outcomes in similar populations but should be interpreted as preliminary and require prospective validation. Notably, in this study, 9 patients (26.5%) remained progression-free at the final follow-up, with a maximum PFS of 20.8 months. Moreover, Kaplan-Meier curves showed that patients with none or small amount of ascites exhibited significantly prolonged PFS compared to those with moderate amount of ascites (10.7 vs. 5.5 m, *p* < 0.05).

Ascites represents a cardinal manifestation in GC patients with PM. Effective ascites management is crucial for alleviating the symptom burden and improving quality of life in this population. Our results showed that HIPEC combined with PD-1 inhibitor and systemic chemotherapy showed significant effectiveness in managing ascites and lowering recurrence rates in patients with GC, aligning with findings from another study (15). In the group of patients who did not have ascites at the beginning of the study (n = 16), a single treatment cycle successfully prevented the onset of new ascites. In the subgroup of patients with moderate amount of ascites (n = 18), the first treatment cycle demonstrated significantly superior efficacy in ascites control compared to the second cycle (HR = 54.70, 95% CI: 7.417-403.4, *p* < 0.0001). This finding underscores the necessity for regular surveillance and timely clinical management in this population. Notably, 5 patients (27.8%) with moderate amount of ascites experienced complete remission of ascites after a cycle of treatment, there was no documented progression at the final follow-up. This indicates that the therapeutic regimen can improve the quality of life for patients and create opportunities for subsequent therapeutic interventions. Although time to ascites control serves as an exploratory endpoint that reflects symptomatic improvement, its long-term clinical relevance should be interpreted in conjunction with survival outcomes.

Remarkably, three patients experienced significant tumor reduction after just one cycle of HIPEC, as verified by imaging within three months. And these patients subsequently underwent radical gastrectomy, and during the intraoperative examination, no residual peritoneal nodules were found. The postoperative pathological staging for these patients ranged from IIIA to IIIC. It highlighted the potential of this regimen to facilitate conversion therapy in select patients. Moreover, two of three HER2-positive patients underwent radical gastrectomy following the treatment. A third HER2-positive patient with baseline massive ascites experienced complete resolution of the ascites and a notable decrease in tumor markers after just one treatment cycle. This state of remission was sustained until the most recent follow-up, which took place 23.9 months later. It is suggested that HER2-positive patients may represent an advantageous subgroup benefiting from this regimen. Further expansion of the number of cases is needed to explore the relevance.

To identify potential beneficiaries of the combination therapy, we evaluated clinicopathological variables and systemic inflammatory indices, including NLR, PLR, CRP, and SII. Systemic inflammation, which is a recognized factor in the development and spread of tumors, is associated with the prognosis of various solid cancers, including breast, pancreatic, colorectal, and GC (21–24). Several studies have found that preoperative NLR, PLR, and mean platelet volume (MPV) can independently predict OS in PM patients undergoing CRS and HIPEC (25, 26). Despite the lack of statistical significance, elevated levels of these systemic inflammatory markers (NLR, PLR, SII) showed a consistent trend toward worse prognosis.

Furthermore, ascites volume serves as a key indicator of tumor burden and peritoneal disease extent, and was inversely associated with prognosis. In our cohort, a moderate amount of ascites was identified as a risk factor for disease progression in univariable analysis. Univariable Cox regression analysis showed that younger age (≤ 60 years) was also associated with poorer survival outcomes. This may be attributable to a more aggressive tumor biology characterized by heightened invasiveness and metastatic potential in younger patients (27). These findings underscore the need for more intensive treatment strategies and vigilant follow-up in this patient population. Collectively, age and ascites volume represent critical biomarkers for stratifying patients who are more likely to benefit from this combination therapy. These findings should be considered exploratory and require validation in larger, prospective studies with multivariable adjustment.

This study has several limitations. First, the small sample size (n=34) restricts statistical power and precludes multivariable analysis to adjust for potential confounders. Residual confounding, such as imbalances in tumor biology, treatment intensity, or biomarker status, may influence the observed associations. Additionally, it is well established that microsatellite instability-high (MSI-H) status, tumor mutational burden (TMB), and PD-L1 expression are key predictive biomarkers for immunotherapy response (28). Nevertheless, their association with patient prognosis could not be assessed in this study because of the limited sample size and substantial missing data. Therefore, the findings of this study should be considered preliminary and exploratory. Further large-scale, sufficiently powered prospective studies are warranted to validate these results. Nonetheless, our study confirms the potential efficacy of this novel therapeutic regimen in GC patients with PM.

## 5 Conclusion

In this retrospective single-center cohort, the combination of HIPEC with PD-1 inhibitor and systemic chemotherapy showed encouraging survival and ascites control with an acceptable safety profile; prospective controlled trials are needed.

## Data Availability

The raw data supporting the conclusions of this article will be made available by the authors, without undue reservation.
