# Supplementary material for: The clinical significance of hyperthermic intraperitoneal chemotherapy combined with PD-1 inhibitor and systemic chemotherapy for advanced gastric cancer patients with peritoneal metastasis: a single-center retrospective study
Source: Front Oncol. 2026 Jan 12;15:1728724. doi: 10.3389/fonc.2025.1728724 (PMC12832277; doi:10.3389/fonc.2025.1728724)
Supplement: Supplementary file 1 [file DataSheet1.docx]

**Supplementary Table S1. PD-L1 expression and HER2 status of the 34 patients**

| Patient ID | PD-L1 CPS | HER2 Status |
| --- | --- | --- |
| 1 | ＜1 | Negative |
| 2 | ＜1 | Negative |
| 3 | 5 | Negative |
| 4 | 1 | Negative |
| 5 | ＜1 | Negative |
| 6 | ＜1 | Negative |
| 7 | ＜1 | Negative |
| 8 | Unknown | Negative |
| 9 | 1 | Positive (IHC 3+) |
| 10 | Unknown | Negative |
| 11 | ＜1 | Negative |
| 12 | ＜1 | Negative |
| 13 | Unknown | Negative |
| 14 | 3 | Negative |
| 15 | ＜1 | Positive (IHC 3+) |
| 16 | 5 | Negative |
| 17 | 5 | Negative |
| 18 | 10 | Negative |
| 19 | 3 | Negative |
| 20 | ＜1 | Negative (IHC 2+/FISH-) |
| 21 | ＜1 | Negative |
| 22 | 60 | Negative (IHC 2+/FISH-) |
| 23 | 2 | Negative (IHC 2+/FISH-) |
| 24 | ＜1 | Positive (IHC 3+) |
| 25 | ＜1 | Negative |
| 26 | ＜1 | Negative |
| 27 | Unknown | Negative |
| 28 | Unknown | Negative |
| 29 | ＜1 | Negative |
| 30 | Unknown | Unknown |
| 31 | ＜1 | Negative |
| 32 | Unknown | Negative |
| 33 | Unknown | Negative |
| 34 | ＜1 | Unknown |

**Abbreviations:** CPS, Combined Positive Score; HER2, Human Epidermal Growth Factor Receptor 2; IHC, Immunohistochemistry; FISH, Fluorescence In Situ Hybridization.


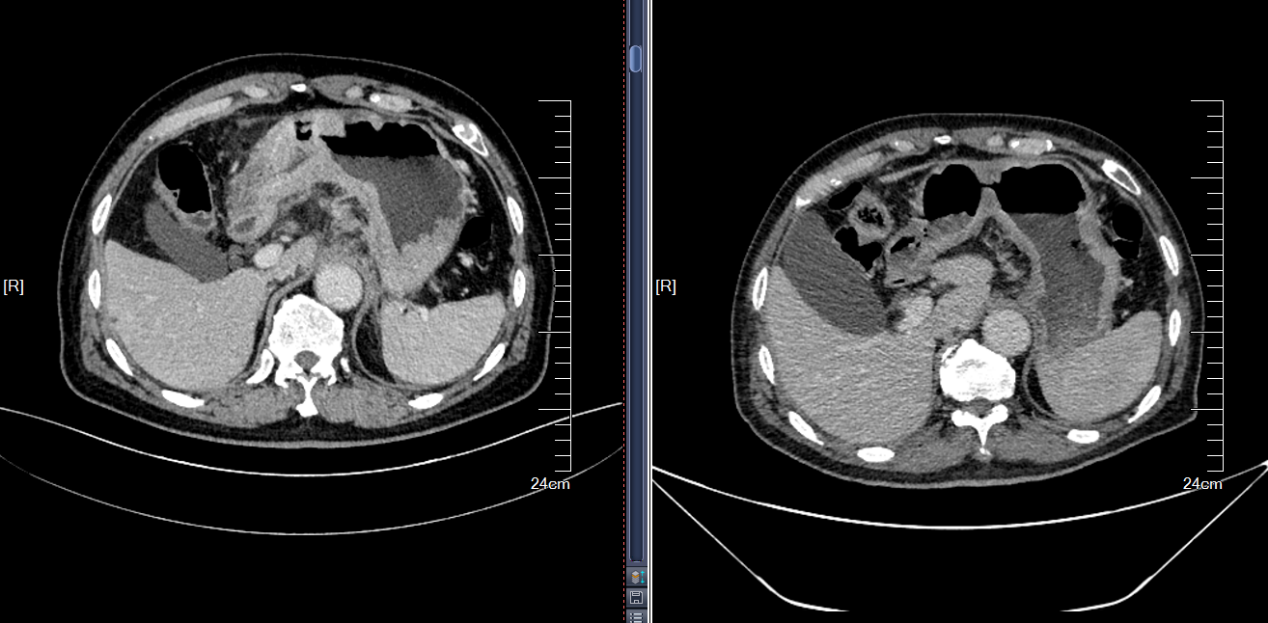
 **Supplementary Figure S1. Representative abdominal CT images before and after treatment in a patient with moderate amounts of ascites.**
Comparison of axial views shows significant shrinkage of the target lesion and reduction in size of retroperitoneal lymph nodes following one cycle of combination therapy.


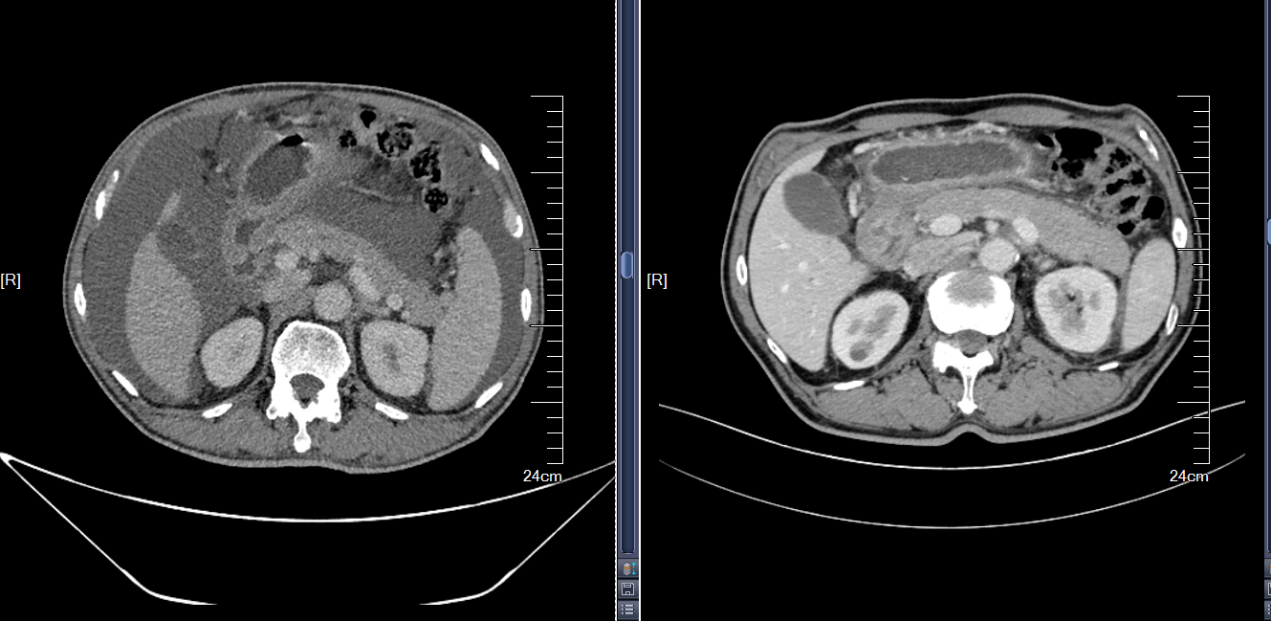


**Supplementary Figure S2. Representative abdominal CT images before and after treatment in another patient.**
Comparison of axial views demonstrates marked reduction of ascites and improvement of peritoneal lesions after one cycle of combination therapy.
